# An FPTAS for Connectivity Interdiction

**DOI:** 10.1007/s10107-025-02312-2

**Published:** 2025-12-16

**Authors:** Chien-Chung Huang, Nidia Obscura Acosta, Sorrachai Yingchareonthawornchai

**Affiliations:** 1 Department of Computer Science, École Normale Supérieure, Paris, France; 2 Department of Computer Science, Aalto University, Espoo, Finland; 3 Institute for Theoretical Studies, ETH Zürich, Zürich, Switzerland

**Keywords:** Approximation Algorithms, Combinatorial Optimization, 68Q25, 05C85, 90C27

## Abstract

In the connectivity interdiction problem, we are asked to find a global graph cut and remove a subset of edges under a budget constraint, so that the total weight of the remaining edges in this cut is minimized. This problem easily includes the knapsack problem as a special case, hence it is NP-hard. For this problem, Zenklusen [Zenklusen’14] designed a polynomial-time approximation scheme (PTAS) and exact algorithms for the special case of unit edge costs. He posed the question of whether a fully polynomial-time approximation scheme (FPTAS) is possible for the general case. We give an affirmative answer. For the special case of unit edge costs, we also give faster exact and approximation algorithms. Our main technical contribution is to establish a connection with an intermediate graph cut problem, called the *normalized* min-cut, which, roughly speaking, penalizes the edge weights of the remaining edges more severely, when more edges are taken out for free.

## Introduction

We study the *connectivity interdiction problem* [1]. In this problem, we are given an instance $(G=(V,E),w,c,b)$, where *G* is an undirected multi-graph which has weights $w:E\rightarrow \mathbb {Z}_{>0}$ and costs $c:E\rightarrow \mathbb {Z}_{>0}$ associated with its edges. A subset of edges $F\subseteq E$ is feasible if $c(F)=\sum _{e \in F}c(e)$ is no more than the budget $b \in \mathbb {Z}_{>0}$. The objective is to remove a feasible set of edges so that in the remaining graph $G'=(V, E\backslash F)$, the minimum weight of a global cut is minimized. Connectivity interdiction arises as a natural optimization problem in graphs. The potential applications include drug delivery intersection [2], nuclear smuggling interdiction [3], security of electric grids under terrorist attacks [4], hospital infection control [5], the vulnerability of the network infrastructure [6], and strategic military planning in the battlefield  [7].

We note that connectivity interdiction can also be regarded as the *b-free min-cut problem*, that is, we aim at finding a vertex set $S \subsetneq V$ and a feasible subset of edges $F \subseteq \delta (S)$ (where $\delta (S)$ is the set of edges with exactly one endpoint in *S*), so that $\sum _{e\in F}c(e) \le b$ and $\sum _{e\in \delta (S)\backslash F}w(e)$ is minimized.

It is easy to see that even in a graph as simple as two vertices connected by parallel edges, the *b*-free min-cut problem already contains the knapsack problem as a special case, and hence is NP-hard. A very natural special case is that of unit edge costs, that is when $c(e) = 1$ for all $e\in E$. This case is known to be in P.

In the rest of this paper, we assume that $n=|V|$ and $m=|E|$. For convenience, we define a cut as the set of edges instead of the set of vertices. That is, we say that a set of edges *C* is a cut if $C = \delta (S)$ for some $S \subsetneq V$. We denote the weight of a cut *C*, as $w(C) = \sum _{e \in C}w(e)$: the total sum of the weight of its edges. Throughout the paper, the notation $\tilde{O}(\cdot )$ hides poly-logarithmic factors in *n* and *m*. Zenklusen [1] has shown the following two results for the connectivity problem.

- A PTAS for arbitrary edge costs.
- For the unit edge costs, an $\tilde{O}(m^2n^4)$ deterministic exact algorithm and an $\tilde{O}(m n^4)$ randomized exact algorithm^1^.

In [1] Zenklusen has posed the open question whether it is possible to obtain an FPTAS with arbitrary edge costs.

### Our results and techniques

We give an affirmative answer to Zenklusen’s question, showing an FPTAS for arbitrary edge costs. Let $(G=(V,E),w,c,b)$ be an instance of the *b*-free min-cut problem.

#### Theorem 1

(FPTAS) With arbitrary edge costs, there is a fully polynomial time approximation scheme for the *b*-free min-cut problem: given $\delta >0$, in time $O(\frac{m^2 n^4}{\delta } \log (n w_{max}b))$, where $w_{\max } = \max _{e \in E}w(e)$, we can find an $(1+\delta )$-approximate solution.

For the special case of unit edge costs, we also improve on the running time of Zenklusen. We design three algorithms that offer varying trade-offs between the running time and the accuracy.

#### Theorem 2

With unit edge costs and for positive accuracy parameter $\varepsilon < 1/100$, we can

1. find an exact solution in $\tilde{O}(m+n^4 b)$ time;
2. or find an $(1+\varepsilon )$-approximate solution in $\tilde{O}(\frac{m}{\varepsilon } +n^3 b)$ time;
3. or find an $(2+\varepsilon )$-approximate solution in $\tilde{O}(\frac{m}{\varepsilon } + n^2 b)$ time.

All these algorithms are randomized and succeed with high probability.

***Our Proof Method.***

We first present the intuition behind our approach. For simplicity of exposition, let us assume an oracle that supports the following query: Given a cut *C*, it returns an optimal subset $F \subseteq C$ to interdict, i.e., $F \subseteq C,\sum _{e \in F}c(e) \le b$ and $\sum _{e \in C \setminus F} w(e)$ is minimized. This problem is known as the knapsack minimization problem, where an FPTAS is known [9]. Given this oracle, how do we obtain an $(1+\varepsilon )$-approximate solution? Trivially, one can enumerate all possible cuts and feed each cut to the oracle, but this takes exponential time as the number of cuts is exponential.

Our key innovation is that we can *reweight* edges of the graph so that a near-optimal interdiction can be found by calling an oracle to one of the approximate mincuts that can be enumerated efficiently. Since the number of approximate cuts is polynomial, the number of calls to the oracle becomes polynomial. More precisely, let positive $\varepsilon < 1/100$ be an accuracy parameter. Let $\alpha _1 = 2+2\varepsilon ,\alpha _2 = 2 -4\varepsilon ,\alpha _3 = 1.5 -2\varepsilon$. We say that a cut *C* is an $\alpha$-approximate cut if the weight of *C* is at most $\alpha$ factor times the value of a global min-cut. The key approach is to show the following.

#### Theorem 3

With arbitrary costs, we can re-define a weight function $\tilde{w}^{(i)}$ where $i \in \{1,2,3\}$ in the same graph *G* so that at least one $\alpha _i$-approximate min-cut *C* w.r.t. $\tilde{w}^{(i)}$ contains a feasible edge-set $F \subseteq C$ such that $w(C\setminus F)$ is $\beta _i$-approximation to the *b*-free min-cut problem where $\beta _1 = 1, \beta _2 = 1+O(\varepsilon ), \beta _3 = 2+O(\varepsilon )$.

The specific values $\alpha _i$’s are derived from a detailed case analysis. Intuitively, the tighter approximation guarantee of the solutions $\beta _i$’s, the higher number of cuts that must be enumerated, which is $O(n^{\lfloor 2\alpha _i \rfloor })$ [11]. The values of $\alpha _i$’s are chosen to be 2.001 and 1.999, and 1.499 (with appropriate value of $\varepsilon$) so that the number of cuts is $O(n^4), O(n^3)$ and $O(n^2)$, respectively and $\beta _i$ becomes $1, 1+O(\varepsilon ),$ and $2+O(\varepsilon )$, respectively.

To design an FPTAS for general connectivity interdiction, we use the weight function^2^$\tilde{w}^{(1)}$ as stated in Theorem 3 for which one of the $\alpha _1$-approximate cut *C* (w.r.t. the weight function $\tilde{w}^{(1)}$) has a feasible set $F \subseteq C$ such that $w(C\setminus F)$ is an optimal solution to the interdiction problem. Then, we enumerate all $\alpha _1$-approximate cuts in $\tilde{O}(n^{\lfloor 2\alpha _1 \rfloor }) = \tilde{O}(n^4)$ time using Nagamochi et. al [8] cut enumeration algorithm. Given a cut, computing the optimal feasible edge-set corresponds to a knapsack minimization problem, where an FPTAS is known [9].

#### Remark 1

Since our algorithm is a polynomial-time reduction to a knapsack minimization problem, this also implies a pseudo-polynomial time algorithm for general connectivity interdiction by using the pseudo-polynomial time algorithm for the knapsack minimization problem [10]. For details, see Remark 2 in Section 3.

For the case of unit edge costs, we could use any $\tilde{w}^{(i)}$ where $i \in \{1,2,3\}$. By Theorem 3, either exact, $1+O(\varepsilon )$-,$2+O(\varepsilon )$-approximate solutions for connectivity interdiction can be found in the enumeration of $\alpha _i$-approximate cuts where $i = 1,2,3$, respectively. By adapting the tree packing theorem [11], we can show that the number of approximate cuts is $O(n^4), O(n^3), O(n^2)$, respectively. Given a cut, the optimal feasible edge set corresponds to the *b* heaviest edges in the cut w.r.t. *w* (since the cost is uniform).

Here as our goal is to have really fast algorithms; it would be inefficient to “explicitly” list all the $\alpha _i$-approximate cuts and then sum up the edge weights after removing the heaviest *b* edges. To do so, we design a data structure that can output the value $w(C \setminus F)$ quickly. Such a data structure uses several ideas from a recent work of Chekuri and Quanrud [12] (cf. [11]). They introduced the data structures for enumerating and evaluating the weights of $O(n^2)$ many $(1+\epsilon )$-approximate cuts. We extend their framework to enumerate and evaluate the weights of $O(n^3)$ and $O(n^4)$ approximate cuts. To evaluate $w(C \setminus F) = w(C) - w(F)$ quickly, we use priority queues on top of their data structures to evaluate *w*(*F*) in $\tilde{O}(b)$ time. This explains the terms $\tilde{O}(n^4b),\tilde{O}( n^3b), \tilde{O}(n^2b)$, respectively in Theorem 2.

***Normalized min-cut.***

The proof of Theorem 3 hinges on an intermediate problem that we call *the normalized min-cut problem*, defined as follows.

#### Definition 1

(Normalized min-cut) Given (*G*, *w*, *c*, *b*) (originally an instance of a *b*-free min-cut problem), here the objective is to find a cut *C*, and a subset of its edges $F \subset C$ satisfying $0 \le c(F) \le b$, so that $\frac{w(C \backslash F)}{\delta _{c(F)}}$ is minimized where $\delta _i := b - i +1$.

Intuitively, the cost of a cut is “normalized” according to the total cost of edges that are taken out: the less costly the subset *F* taken out, the more heavily the rest of the edges $C\backslash F$ would be normalized.

In fact, originally, the normalized min-cut problem arises in a different (but related) context. In a recent paper of Chalermsook et el. [13] on survivable network design, the same problem was first introduced (under a different name “minimum normalized free cut”) to deal with a certain boxing constraint in the LPs. There, a special case of unit-edge costs is actually *solved as a technical necessity*. To obtain an FPTAS in this paper, we emphasize that we do not need to solve the normalized min-cut problem per se, but rather we use its optimal solution as a certificate in the analysis of the weight function $\tilde{w}^{(i)}$ in Theorem 3. We introduce another notation to facilitate our discussion.

#### Definition 2

For each $0 \le i \le b$, let $\lambda _i = \min _{C, F\subseteq C, c(F)=i} w(C \backslash F)$. (In case that there is no *C* and $F \subseteq C$ so that $c(F) = i$, $\lambda _i$ is understood to be infinity.)

Furthermore, let ${\textsf{OPT}}$ and ${\textsf{OPT}}^N$ denote the value of the optimal solution for the *b*-free min-cut and the normalized min-cut, repsectively. Then by definition,

$${\textsf{OPT}}= \min _{0\le i \le b}\lambda _i, \ \ \ \ {\textsf{OPT}}^N = \min \{\frac{\lambda _0}{b+1}, \frac{\lambda _1}{b}, \ldots , \frac{\lambda _i}{b-i+1}, \ldots , \frac{\lambda _{b-1}}{2},\frac{\lambda _b}{1}\}.$$

Throughout the article, we assume that there is no cut *C* with $w(C) \le b$. Thus, when *c* is unit edge costs, ${\textsf{OPT}}=\lambda _b$; but for arbitrary costs this might not be true.

For both *b*-free min-cut and normalized min-cut, a solution involves a cut *C* and its feasible set of edges $F\subseteq C$. In our presentation, we often write them as a pair (*C*, *F*).

We now explain how the normalized min-cut problem comes into the picture. To define the intermediate weight function, we *truncate* the weights as follows.

#### Definition 3

Let $\tau$ be a parameter. Define a global min-cut problem $(G=(V,E), \tilde{w}_{\tau })$ by truncating the weights *w* as follows: $$\tilde{w}_{\tau }(e) = \left\{ \begin{array}{ c l } \tau \cdot c(e) &  \quad {\text {if }} w(e) \ge \tau \cdot c(e), \\ w(e) &  \quad {\text {otherwise.}} \end{array} \right. $$

An edge $e \in E$ is *heavy* if $w(e) \ge \tau \cdot c(e)$. Otherwise, it is *light*.

Assuming that we are given an estimate $\tau$ of ${\textsf{OPT}}^N$ (we will explain later how such guesses can be made and how to bound the number of such guesses). The following relations of the various optimal cuts (of different problems) is crucial.

#### Theorem 4

(Main) Let $(C^N,F^N)$ be the solution that *realizes* ${\textsf{OPT}}^N$ in the normalized min-cut problem, i.e., ${\textsf{OPT}}^N =\frac{w(C^N \backslash F^N)}{b-c(F^N)+1}$. Similarly, let $(C^*,F^*)$ be a solution that realizes ${\textsf{OPT}}$ in the *b*-free min-cut problem, i.e., $w(C^*\setminus F^*) = {\textsf{OPT}}$.

Suppose that ${\textsf{OPT}}^N(1-\varepsilon ) \le \tau \le {\textsf{OPT}}^N$ where $0<\varepsilon \le 1/100$. Consider the instance $(G,\tilde{w}_{\tau })$ of the global min-cut problem, and denote $C^{\min }$ as an optimal solution.

Finally, let $\alpha _1 = 2+2\varepsilon , \alpha _2 = 2-4\varepsilon ,$ and $\alpha _3 = 1.5-2\varepsilon$. Then the following facts hold.

(i) $\tilde{w}_{\tau }(C^*) \le \alpha _1 \cdot \tilde{w}_{\tau }(C^{\min })$. That is, $C^*$ is $\alpha _1$-approximate min-cut w.r.t. $\tilde{w}_\tau$.
(ii) $\tilde{w}_{\tau }(C^N) \le \alpha _2 \cdot \tilde{w}_{\tau }(C^{\min })$. Furthermore, if $\tilde{w}_{\tau }(C^*) > \alpha _2 \cdot \tilde{w}_{\tau }(C^{\min })$, then $(C^N,F^N)$ is an $(1+O(\varepsilon ))$-approximate solution to the *b*-free min-cut problem.
(iii) $\tilde{w}_{\tau }(C^N) \le \alpha _3 \cdot \tilde{w}_{\tau }(C^{\min })$. Furthermore, if $\tilde{w}_{\tau }(C^*) > \alpha _3 \cdot \tilde{w}_{\tau }(C^{\min })$, then $(C^N,F^N)$ is an $(2+O(\varepsilon ))$-approximate solution to the *b*-free min-cut problem.

Theorem 4 immediately implies Theorem 3, and gives us a trade-off in terms of the number of cuts to enumerate and the quality of the solutions from the normalized min-cut problem. Namely, the number of approximate cuts w.r.t. $\tilde{w}$ to enumerate is $O(n^{\lfloor 2\alpha _1\rfloor }), O(n^{\lfloor 2\alpha _2\rfloor }), O(n^{\lfloor 2\alpha _3\rfloor })$, which is $O(n^4), O(n^3), O(n^2)$, respectively, in order to obtain exact, $(1+O(\varepsilon ))$ and $(2+O(\varepsilon ))$ approximate solutions respectively. To remove the assumption, ${\textsf{OPT}}^N(1-\varepsilon ) \le \tau \le {\textsf{OPT}}^N$, we guess the value of $\tau$ by the geometric series $(1+\varepsilon )^i$ in an appropriate range.

Before finishing, we briefly discuss the difference of our approach with that of Zenklusen. As stated above, ours is based on introducing an intermediate problem and a proper truncating of the weight function. Zenklusen’s approach relies on an intelligent “guessing” strategy. Assume that $(C^*,F^*)$ is a solution that realizes ${\textsf{OPT}}$ in the *b*-free min-cut problem. He guesses not only the least efficient edge in the free set $F^*$ (i.e., $\mathop {\mathrm {arg\,min}}\limits _{e \in F^*}\frac{w(e)}{c(e)}$), but also the $1/\varepsilon$ heaviest edges in $C^*\backslash F^*$. Given a guess, the graph is modified: some edges have their costs modified to 0 while the others have their weights modified to 0. In this new instance, finding a cut *C* that minimizes the weight *w*(*C*) while respecting $c(C) \le b$ will give the desired approximate solution in the original instance. Such a cut can be found by an algorithm of Armon and Zwick [14].

This approach crucially relies on the fact that the heaviest $1/\varepsilon$ edges in $C^*\backslash F^*$ are guessed. In fact such a strategy is often employed for designing algorithms for a variety of knapsack-type problems—but this unavoidably requires $m^{1/\varepsilon }$ guesses, thus precluding an FPTAS. Our approach avoids this onerous step.

***Other Related Work.***

For many optimization problems, one can introduce a corresponding interdiction problem. See [15] for a recent survey on the interdiction problem in general. Roughly speaking, in the interdiction problem, there is a certain “interdictor”, who tries to worsen the optimal value of the optimization problem as much as possible. The present work focuses on the global cut problem. But in fact, a large variety of classical optimization problems have been studied from the point of view of interdiction. Examples include: shortest paths [16–19], network design [20, 21], minimum spanning tree [22], graph matching [23], clustering [24], and network flows [25].

### Organization

In Section 2, we establish the connections between the normalized min-cut and the *b*-free min-cut problems, which allows us to prove our main technical result, Theorem 4. In Section 3, we show our FPTAS for general edge costs; furthermore, in Section 3.1, we discuss how our approach can be extended to design an FPTAS for the more general *b*-free *k*-cut problem. In Section 4 we show our algorithms for unit edge costs.

## The cormalized min-cut problem

We prove Theorem 4 in this section.

***Proof Outline.***

We first show the two structural properties of the optimal solution $(C^N,F^N)$ to the normalized min-cut problem: (1) every $e \in F^N$ is heavy, i.e., every edge in the optimal free-edge set is heavy, and (2) $C^N$ is an $(1+2\varepsilon )$-approximate cut in the new weight function $\tilde{w}_{\tau }$ (Definition 3). Next, we prove that if $C^*$ is not $\alpha$-approximate cut in $\tilde{w}_{\tau }$, then we can establish a good lower bound on ${\textsf{OPT}}$ in the original weight function *w*. This allows us to show that the optimal solution to the normalized min-cut $(C^N,F^N)$ is a good approximate solution to the *b*-free min-cut problem. Depending on the parameter $\alpha$, we obtain different guarantees as shown in Theorem 4. We now formalize the proofs.

***Structures of*** $$(C^N,F^N)$$ ***.***

The following fact will be useful.

### Lemma 1

Suppose that ${\textsf{OPT}}^N$ is realized by $(C^N,F^N)$, namely, ${\textsf{OPT}}^N =\frac{w(C^N\backslash F^N)}{\delta _{c(F)}}$. Then every edge $e \in F^N$ is a heavy edge.

### Proof

Suppose, for a contradiction, that $e \in F^N$ is a light edge. Then $w(e) < \tau c(e)$. Let $F' = F^N \backslash \{e\}$. Then

$$ \frac{w(C^N \backslash F')}{\delta _c(F')} = \frac{w(C^N \backslash F^N)+ w(e)}{\delta _{c(F^N)}+ c(e)} < \frac{w(C^N \backslash F^N)+ \tau c(e)}{\delta _{c(F^N)}+ c(e)} $$

$$ \le \frac{w(C^N \backslash F^N)+ (\frac{w(C^N \backslash F^N)}{\delta _{c(F^N)}})c(e)}{\delta _{c(F^N)}+ c(e)} = \frac{w(C^N \backslash F^N)}{\delta _{c(F^N)}} $$

where in the second inequality we use the fact that $\tau \le {\textsf{OPT}}^N$. As $c(F') \le c(F^N) \le b$, we have obtained a contradiction, since the pair $(C^N, F')$ reaches a value smaller than ${\textsf{OPT}}^N$. $\square$

A main subroutine of our algorithms is to enumerate all $\alpha$-approximate cuts in the global cut instance $(G, \tilde{w}_{\tau })$. We will first establish a lower bound on the optimal value of the global cut in the latter.

### Lemma 2

Given any cut *C* in $(G, \tilde{w}_{\tau })$, then $\tilde{w}_{\tau }(C) \ge \tau (1+ b)$.

### Proof

Let $F \subseteq C$ be the set of heavy edges of *C*. Suppose that $c(F) \ge b+1$. Then $\tilde{w}_{\tau }(C) \ge \tilde{w}_{\tau }(F) \ge \tau c(F) \ge \tau (1 + b).$ On the other hand, suppose that $c(F)=i < b+1$. Then

$$\tilde{w}_{\tau }(C) = \tilde{w}_{\tau }(F)+ \tilde{w}_{\tau }(C\backslash F) = i \tau + w(C \backslash F) \ge i \tau + {\textsf{OPT}}^N \delta _i \ge i \tau + \tau \delta _i = \tau (1 + b),$$

where in the second inequality we use the fact that $w(C\backslash F) \ge \lambda _i \ge {\textsf{OPT}}^N \delta _i$. $\square$

### Lemma 3

Suppose that ${\textsf{OPT}}^N$ is realized by $(C^N,F^N)$. Furthermore, $C^{\min }$ is the optimal global cut in the instance $(G, \tilde{w}_{\tau })$. Then

$$\frac{\tilde{w}_{\tau }(C^N)}{\tilde{w}_{\tau }(C^{\min })} < 1 + 2\varepsilon ,$$

assuming that $$\varepsilon <1/2$$.

### Proof

By Lemma 2, we already know that $\tilde{w}_{\tau }(C^{\min }) \ge \tau (1+ b)$. It remains to upper bound $\tilde{w}_{\tau }(C^N)$. By Lemma 1, all edges in $F^N$ are heavy. Thus $\tilde{w}_{\tau }(F^N) = \tau c(F^N)$. Furthermore, as $\tilde{w}_{\tau }(C^N \backslash F^N) \le w(C^N \backslash F^N) = \delta _{c(F^N)}{\textsf{OPT}}^N$. Therefore

$$\tilde{w}_{\tau }(C^N)\le \tau c(F^N) + \delta _{c(F^N)}{\textsf{OPT}}^N \le \tau c(F^N) + \frac{\tau }{1-\varepsilon } \delta _{c(F^N)} $$

$$ \le \frac{\tau }{1-\varepsilon }(c(F^N) + \delta _{c(F^N)}) = \frac{\tau }{1-\varepsilon }(1+ b).$$

In conclusion, we have $\frac{\tilde{w}_{\tau }(C^N)}{\tilde{w}_{\tau }(C^{\min })}< 1/(1 -\varepsilon ) < 1+2 \varepsilon$. $\square$

***Lower bound on*** ${\textsf{OPT}}$ ***.***

The next lemma states that if after enumerating all $\alpha$-approximate cuts in $(G, \tilde{w}_{\tau })$, we still cannot find an optimal solution in the original *b*-free min-cut instance, we can then establish a lower bound on ${\textsf{OPT}}$.

### Lemma 4

Let $C^*$ denote the optimal solution in the *b*-free min-cut instance (*G*, *w*, *c*, *b*). Namely, there exists $F^* \subseteq C^*$ so that $c(F^*)\le b$ and $w(C^* \backslash F^*) = {\textsf{OPT}}$. Then either $C^*$ is an $\alpha$-approximate cut in the instance $(G, \tilde{w}_{\tau })$, for some $\alpha \ge 1$, or ${\textsf{OPT}}\ge \tau (\alpha + (\alpha -1)b)$.

### Proof

Suppose that $C^*$ is not an $\alpha$-approximate cut in $(G,\tilde{w}_{\tau })$. Let $C^{\min }$ denote the optimal global cut in $(G,\tilde{w}_{\tau })$. Then

$$\alpha \le \frac{\tilde{w}_{\tau }(C^*)}{\tilde{w}_{\tau }(C^{\min })}\le \frac{\tilde{w}_{\tau }(F^*) + \tilde{w}_{\tau }(C^*\backslash F^*)}{\tau (1+ b)} \le \frac{c(F^*)\tau + w(C^*\backslash F^*) }{\tau (1+ b)} \le \frac{b\tau + {\textsf{OPT}}}{\tau (1+ b)}, $$

where the second inequality uses Lemma 2. $\square$

### Corollary 1

Suppose that ${\textsf{OPT}}^N$ and ${\textsf{OPT}}$ are realized by $(C^N,F^N)$ and $(C^*, F^*)$ respectively, namely, ${\textsf{OPT}}^N =\frac{w(C^N\backslash F^N)}{\delta _{c(F^N)}}$, and $w(C^* \backslash F^*) = {\textsf{OPT}}$. Furthermore, $C^*$ is not an $\alpha$-approximate cut in $(G, \tilde{w}_{\tau })$ for some $\alpha \ge 1$. Then

$$ \frac{w(C^N \backslash F^N)}{w(C^* \backslash F^*)} \le \frac{1+ b}{(1-\varepsilon )(\alpha + (\alpha -1)b)}. $$

### Proof

$$\frac{w(C^N \backslash F^N)}{w(C^* \backslash F^*)} =\frac{{\textsf{OPT}}^N \cdot \delta _{c(F^N)}}{{\textsf{OPT}}} \le \frac{{\textsf{OPT}}^N \cdot \delta _{c(F^N)}}{\tau (\alpha + (\alpha -1)b)} $$

$$ \le \frac{\frac{\tau }{1-\varepsilon }(1+ b-c(F^N))}{\tau (\alpha + (\alpha -1)b)} \le \frac{1+ b}{(1-\varepsilon )(\alpha + (\alpha -1)b)}, $$

where the first inequality uses Lemma 4. $\square$

***Finishing the proof.***

We are now ready to prove Theorem 4. For each item of the statement, we need to set an appropriate value of $\alpha$. We next show that by setting $\alpha$ to be suitably large, the optimal solution in *b*-free min-cut must be an $\alpha$-approximate cut in $(G, \tilde{w}_{\tau })$—this leads to an FPTAS for non-uniform cost and an exact algorithm for uniform costs.

### Theorem 5

Suppose that $\alpha = 2 + 2\varepsilon$, with $0 <\varepsilon \le 1/5$. Then the optimal cut $C^*$ that realizes ${\textsf{OPT}}$ must be $\alpha$-approximate cut in $(G, \tilde{w}_{\tau })$.

### Proof

For a contradiction, suppose that $C^*$ is not $\alpha$-approximate in $(G, \tilde{w}_{\tau })$. Then by Corollary 1,

$$\frac{w(C^N \backslash F^N)}{w(C^* \backslash F^*)} \le \frac{1+ b}{(1-\varepsilon )(\alpha + (\alpha -1)b)}<1,$$

where the last inequality can be easily verified. Then we reach a contradiction that $C^N \backslash F^N$ is an even cheaper solution than $C^*\backslash F^*$. $\square$

### Proof of Theorem 4

We first prove Part 1. Let $\alpha = 2 + 2\varepsilon$ with $\varepsilon = 0.01$. Theorem 5 already gives us that the optimal cut $C^*$ which realizes ${\textsf{OPT}}$ must be a $2 + 2\varepsilon$-approximate cut in $(G, \tilde{w}_{\tau })$ as desired.

We next proceed to prove Parts 2 & 3. First note that by Lemma 3, $\tilde{w}_{\tau }(C^N) \le \alpha \cdot \tilde{w}_{\tau }(C^{\min })$ for $\alpha = 2 - 4\varepsilon$ and $\alpha = 1.5 - 2\varepsilon$.

Let $\alpha = 2 - 4\varepsilon$, note that contrary to Part 1, we cannot ensure that the cut $C^*$ which obtains ${\textsf{OPT}}$ will be a $2-4\varepsilon$-approx cut in $(G, \tilde{w}_{\tau }$). However, we know that by Corollary 1 the cut $C^N$ achieving the optimal value in the normalized mincut problem is an approximation to the optimum *b*-free min cut as follows:

$$ \frac{w(C^N \backslash F^N)}{w(C^* \backslash F^*)} \le \frac{1+ b}{(1-\varepsilon )(\alpha + (\alpha -1)b)} = \frac{1+ b}{(1-\varepsilon )((2 - 4\varepsilon ) + (1 - 4 \varepsilon )b)} $$

$$ \le \frac{1}{(1 - \varepsilon )(1 - 4 \varepsilon )} \le 1 + 8\varepsilon . $$

For $\alpha = \frac{3}{2} - 2\varepsilon$ we have:

$$\frac{w(C^N \backslash F^N)}{w(C^* \backslash F^*)} \le \frac{1+ b}{(1-\varepsilon )((\frac{3}{2} - 2\varepsilon ) + (\frac{1}{2} - 2 \varepsilon )b)} \le \frac{1}{(1 - \varepsilon )(\frac{1}{2} - 2 \varepsilon )} \le 2 + 14\varepsilon .$$

To see the last inequality, observe that $1/(1-\varepsilon )(1/2-2\varepsilon )=2/(1-5\varepsilon + 4\varepsilon ^2) \le 2/(1-5\varepsilon ) \le 2+14\varepsilon$, where the last inequality uses $\varepsilon \le 0.01$. $\square$

## An FPTAS for general connectivity interdiction

We explain how to obtain an FPTAS with arbitrary edge costs, proving Theorem 1.

### Theorem 6

Let $(C^*,F^*)$ be an optimal *b*-free min-cut of the instance $(G = (V,E),w,c,b)$. There is a weight function $\tilde{w}^*: E \rightarrow \mathbb {R}_{\ge 0}$ such that $C^*$ is a (2.01)-approximate min-cut w.r.t. $\tilde{w}^*$. Furthermore, there is an efficient algorithm that outputs a collection $\mathcal {W}$ of weight functions such that (1) $\mathcal {W}$ contains such a function $\tilde{w}^*$ and (2) $|\mathcal {W}| = O(\log (m\cdot w_{\max } \cdot b))$.

Theorem 6 follows from Theorem 4(i) by guessing the appropriate value of $\tau$ in the range of $(1+\varepsilon )^i$ for some fixed $\varepsilon$. For each guess $\tau$, we define the weight function as in Definition 3 according to $\tau$. We defer the proof to the end of the section.

### Proposition 7

Given a cut *C*, suppose that $F\subseteq C$ is the optimal feasible edge-set, then we can compute, in $O(m^2/\delta )$ time for $\delta >0$, a feasible edge-set $F'$ so that $c(F') \le b$ and $w(C \backslash F') \le (1+\delta ) w(C \backslash F).$

Proposition 7 follows from a simple reduction to knapsack minimization problem for which an FPTAS is known [9].

Furthermore, in [10] a pseudo-polynomial time algorithm running in time $O(n^2W)$ for the knapsack minimization is shown (where $W = \max {w_i}$). As we will see in Remark 2, this allows us to get a pseudo-polynomial time algorithm for the general case of connectivity interdiction. We will prove Proposition 7 at the end of the section.

We are now ready to state the FPTAS. We try all weight functions from Theorem 6. For each weight function, we enumerate all 2.1-approximate cuts w.r.t. $\tilde{w}_{\tau }$. For each cut *C*, we compute $(1+\delta )$-approximate feasible edge-set $F \subseteq C$ using Theorem 7. Finally, we output the best pair of a cut and its free-edge set found so far. The algorithm is summarized in Algorithm 1.

**Algorithm 1:**
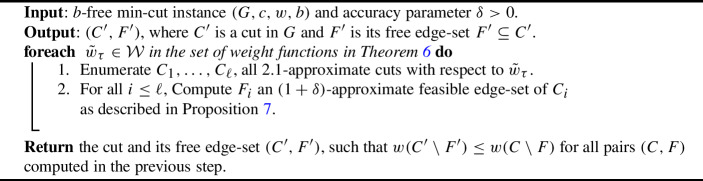
FPTAS for general connectivity interdiction

***Running Time.***

We use Nagamochi et. al [8] cut enumeration algorithm along with the fact that the number of $\alpha$-approximate min-cuts is $O(n^{\lfloor 2\alpha \rfloor }) = O(n^4)$ [11]. We can compute $(1+\delta )$-approximate feasible edge-set in $O(\frac{1}{\delta } \cdot m^2)$ time using Theorem 7. By Theorem 6, we repeat for $|\mathcal {W}| = O( \log (n\cdot w_{\max }\cdot b))$ iterations. Therefore, the algorithm runs in $O(\frac{m^2n^4}{\delta } \log (n w_{max}b))$ time.

***Correctness.***

By Theorem 6, there is an $\tilde{w}^* \in \mathcal {W}$ such that $C^*$ is an $(2+2\varepsilon )$-min-cut w.r.t. $\tilde{w}^*$ for $\varepsilon < 1/100$. Since we enumerate all 2.1-approximate min-cuts in $w^*$, we are bound to encounter $C^*$ at some point in the enumeration. By Proposition 7, we can compute a set $F\subseteq C^*$ so that $w(C^* \backslash F) \le (1+\delta )w(C^* \backslash F^*) \le (1+\delta ){\textsf{OPT}}^*$. As a result, the solution $(C',F')$ returned by the algorithm must be an $(1+\delta )$-approximate solution to the *b*-free min-cut problem.

### Remark 2

Note that to obtain a polynomial time algorithm for the general connectivity interdiction problem when the value $W = \max {w_i}$ is polynomial in the input size, we replace step 2 in algorithm 1 with the pseudo-polynomial time algorithm from [10].

We now prove Theorem 6 and Proposition 7.

### Proof of Theorem 6

Observe that by the definition of ${\textsf{OPT}}^N$, it follows easily that^3^

$$ \frac{1}{b+1} \le {\textsf{OPT}}^N \le \lambda _0.$$

Trivially, $\lambda _0 \le m w_{\max }$. Thus ${\textsf{OPT}}^N \in [\frac{1}{b+1}, m w_{\max }]$. We define the collection of weight functions as follows. Let $\mathcal {W} = \{\}$ and let $\varepsilon \le 1/100$. For all $j=0, 1, \cdots$ so that $\frac{(1+\varepsilon )^j}{b+1} \le mw_{\max }$, set $\tau =\frac{(1+\varepsilon )^j}{b+1}$, and define $\tilde{w}_{\tau }$ according to $\tau$ (as in Definition 3) and add it into $\mathcal {W}$. The number of weight functions is $|\mathcal {W}| = O(\log mw_{\max }b)$; furthermore, one of the $\tau$s must satisfy ${\textsf{OPT}}^N(1-\varepsilon ) \le \tau \le {\textsf{OPT}}^N$ for $\varepsilon \le 1/100$. Therefore, Theorem 4(i) applies to that weight function. $\square$

### Proof of Proposition 7

Finding the optimal set $F^*$ reduces to the following knapsack minimization problem.

(Knapsack Minimization) Find a subset $A^* \subseteq C^*$, which respects $c(A^*) \ge (\sum _{e\in C^*}c(e))-b$, so that $w(A^*)$ is minimized.

$C^*\backslash F^*$ is exactly the optimal solution $A^*$ in the above knapsack problem. It is known [9] that, in $O(m^2/\delta )$ time for $\delta >0$, one can find a solution *A* so that $c(A) \ge (\sum _{e\in E}c(e))-b$ and $w(A) \ge (1+\delta ) w(A^*)$. Setting *F* to be $C^* \backslash A$ gives the desired proof. $\square$

### Extension to generalized connectivity interdiction

We show that our approach can be adapted to a more general version of the connectivity interdiction problem. Given the same input instance $(G=(V,E),w,c,b)$, suppose that we aim at removing a subset $F\subseteq E$, $c(F)\le b$, so that in the remaining graph $G'=(V,E\backslash F)$, the weight of a *k*-*cut* is minimized. We recall that a *k*-cut is a set of edges whose removal splits the graph into at least *k* components.

Equivalently, this problem is the same as the *b*-free *k*-cut problem. We look for a vertex partition $C=(C_1,\cdots , C_k)$ of *V*, where $E(C)\subseteq E$ is the set of edges whose endpoints lie at different $C_i$s, so that there exists $F\subseteq E(C)$, $\sum _{e \in F}c(e)\le b$, and $\sum _{e \in E(C)\backslash F}w(e)$ is minimized. For simplicity, we slightly abuse the notation by writing the set of edges *E*(*C*) as simply *C*, just as in the case of global cut.

It is straightforward to generalize Definitions 1-3 and Theorem 4(i) and Theorem 6 to this *k*-cut setting.^4^ Thus, to reproduce an FPTAS, the only question is that given a weight function $\tilde{w}_{\tau }$, how do we enumerate all the 2.1-approximate *k*-cuts?

The following notion of *r*-*respecting cut* was first introduced by Karger [11].

#### Definition 4

*(r-respecting cut)* Let $G=(V,E)$ be a graph and let *T* be a spanning tree of *G*. We say that a $(k-)$cut *C* in *G* *r*-respects *T*, if $|C\cap E(T)| = r$.

We use the following result of Chekuri et al. [26]. Let $\mathcal {T}(G)$ denote the set of all spanning trees. Below we write the primal and the dual LPs of the *k*-cut problem.
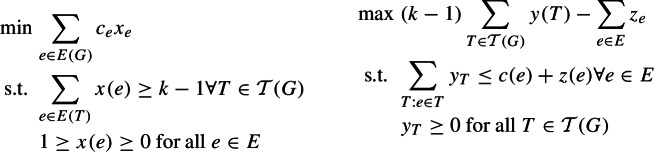


#### Theorem 8

(cf. Lemma 7 of [26], slightly simplified) Let $y^*$, $z^*$ be an optimum solution to the dual LP for *k*-cut. Let *C* be an $\alpha$-approximate *k*-cut for some $\alpha \ge 1$. Given an integer $h\ge k-1$, let $q_h$ be the fraction of the trees *T* in *y* so that $|E(T) \cap C| \le h$. Then

$$q_h \ge 1 - \frac{2\alpha (k-1)(2-\frac{1}{n})}{h+1}.$$

For our application, $\alpha =2.1$, therefore, by setting $h=5k-4$, $q_h >0$, we guarantee that a given $\alpha$-approximate *k*-cut $h'$-respects, $h'\le h$, one of the trees in $y^*$.

We now explain how to enumerate all 2.1-approximate cuts in polynomial time. First, we use the Ellipsoid algorithm [27] to solve the LPs. This guarantees that in the optimal dual solution $y^*$, there are at most *m* trees. For each of the trees *T* and for each $h'$, $k-1 \le h' \le h$, enumerate all the ${n-1 \atopwithdelims ()h'}$ edges in *T*. When $h'$ edges are removed, *T* is split into $h'+1 \ge k$ connected components. These components can be assigned to *k* different parts to define a *k*-cut, in $k^{h'+1}$ ways. In summary, we have $m (nk)^{O(k)}$ possibilites to check, in order to enumerate all 2.1-approximate *k*-cuts.

## Fast algorithms for unit edge costs

We prove Theorem 2 in this section. We assume that the given instance (*G*, *c*, *w*, *b*) has unit edge costs $c:E\rightarrow 1$. Notice that in this setting, once a cut *C* is chosen, the best feasible set of edges $F\subseteq C$ to be removed is simply the *b* heaviest edges according to *w*. We will call these edges the *free set* (of *C*).

### The schematic algorithm

We now describe the algorithm. The inputs consist of a *b*-free mincut instance (*G*, *c*, *w*, *b*) and two positive parameters $\varepsilon < 1/100$ and $\alpha \in \{\alpha _1,\alpha _2,\alpha _3\}$ where $\alpha _1 := 2+2\epsilon ,\alpha _2 := 2-4\epsilon ,\alpha _3 := 1.5 -2\epsilon$. The parameter $\alpha$ controls the trade-off between the approximation ratio and the running time. First, we compute an estimate $\tau$ such that ${\textsf{OPT}}^N(1-\varepsilon ) \le \tau \le {\textsf{OPT}}^N$. Then, we define the weight function $\tilde{w}_\tau$ according to Definition 3. We enumerate all $\alpha$-approximate cuts, and for each cut *C* we compute $w(C \setminus F)$ where *F* is the free set of *C*. Finally, we return the cut $C'$ and its free set $F'$ with the smallest value of $w(C'\setminus F')$ so far. We summarize the algorithm in Algorithm 2.

**Algorithm 2:**
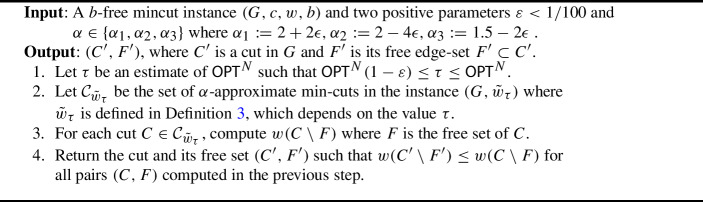
The algorithm for the unit edge costs

***Analysis.***

We next describe the analysis of Algorithm 2.

#### Theorem 9

Let $(C',F')$ be the solution returned by Algorithm 2 on the *b*-free mincut instance (*G*, *c*, *w*, *b*) with parameters $\varepsilon < 1/100$ and $\alpha \in \{\alpha _1,\alpha _2,\alpha _3\}$.

1. If $\alpha = \alpha _1$, and $\varepsilon = 0.001$, then $(C',F')$ is optimal and it can be implemented to run in $\tilde{O}(m+n^4b)$ time.
2. If $\alpha = \alpha _2$, then $(C',F')$ is $(1+O(\varepsilon ))$-approximation and it can be implemented to run in $\tilde{O}(\frac{1}{\varepsilon }\cdot m + n^3b)$ time.
3. If $\alpha = \alpha _3$, then $(C',F')$ is $(2+O(\varepsilon ))$-approximation and it can be implemented to run in $\tilde{O}(\frac{1}{\varepsilon }\cdot m + n^2b)$ time.

Every implementation is randomized and succeeds with high probability.

Theorem 2 follows immediately from Theorem 9. For implementation and running time analysis, we will discuss the necessary tools in Section 4.2. Here we just focus on proving the correctness.

***Correctness.***

We now prove the correctness of the algorithm in Theorem 9. Let $(C^*,F^*)$ be an optimal solution to *b*-free min-cut problem, and $(C^N,F^N)$ be an optimal solution to the normalized min-cut problem. In each case of $\alpha \in \{\alpha _1,\alpha _2,\alpha _3\}$, we apply Theorem 4 using $\tilde{w}_{\tau }$ as defined in Definition 3, where ${\textsf{OPT}}^N(1-\epsilon ) \le \tau \le {\textsf{OPT}}^N$.

1. If $\alpha = \alpha _1$ and $\epsilon = 0.001$, then $\mathcal {C}_{\tilde{w}_{\tau }}$ contains $C^*$, and thus $F^*$ was computed in step 3 of the algorithm. Therefore, at the end we have $w(C' \setminus F') \le w(C^*\setminus F^*)$.
2. If $\alpha \in \{\alpha _2,\alpha _3\}$, then we consider two different cases depending on $\tilde{w}_{\tau }(C^*)$. If $\tilde{w}_{\tau } (C^*) \le \alpha \tilde{w}_{\tau }(C^{\min })$, where $C^{\min }$ is global min-cut w.r.t. $\tilde{w}_{\tau }$, then $\mathcal {C}_{\tilde{w}_{\tau }}$ contains $C^*$, and then we are done by the same argument in as the previous case. Otherwise, by Theorem 4, $\tilde{w}_{\tau }(C^N)$ is $\alpha$-approximate cut in $\tilde{w}_{\tau }$. Thus, $C^N \in \mathcal {C}_{\tilde{w}_{\tau }}$. In this case, $F^N$ was also computed in step 3 of the algorithm. Therefore, if $\alpha = \alpha _2$, then $w(C' \setminus F') \le w(C^N \setminus F^N) \le (1+O(\varepsilon )) w(C^* \setminus F^*)$, where the last inequality follows from Theorem 4(ii). If $\alpha = \alpha _3$, then $w(C'\setminus F') \le w(C^N\setminus F^N) \le (2+O(\varepsilon )) w(C^* \setminus F^*)$, where the last inequality follows from Theorem 4(iii).

### Fast implementation

For the fast implementation of Algorithm 2, we need an efficient enumeration of $\alpha$-approximate cuts and the efficient evaluation of weights of cuts when their *b* heaviest edges are removed. We achieve the enumeration via the adaptation of the tree packing theorem by Karger [11] for 3 and 4-respecting cuts.

#### Cut enumeration

For the evaluation of cuts with respect to the original weight *w* when their *b*-heaviest edges are removed, we generalize a cut oracle data structure from [12] that *efficiently* computes the weight of 1 and 2-respecting cuts, to also handle 3 and 4-respecting cuts and to handle the computation of their *b* heaviest edges via the addition of a max-heap data structure to cut oracle.

We note that given a spanning tree *T*, each set of *r* tree edges in *T* *induces* a unique cut in *G*. This is indeed the entire base upon which the fast min-cut algorithm of Karger [11] is built.

Karger [11] (see also the discussion [12]) gave a randomized algorithm to compute a collection of $O(\log n)$ spanning trees such that with high probability every $\alpha$-approximate min-cut *r*-respects some spanning tree in the collection.

##### Theorem 10

(Tree Packing Theorem [11] (Restated)) Let $G = (V,E,w)$ be an undirected weighted graph. There is an $\tilde{O}(m)$-time algorithm that takes *G* and a parameter $\alpha$ (where $\alpha \in \{\alpha _1,\alpha _2,\alpha _3\}$, and $\varepsilon < 1/100$) as input and outputs a collection $\mathcal {T}$ of $O(\log n)$ spanning trees (the constant factor in $O(\cdot )$ depends on $\alpha$) satisfying the following with high probability:

- If $\alpha = \alpha _1$, then every $\alpha _1$-approx. min-cut 4-respects a spanning tree in $\mathcal {T}$,
- If $\alpha = \alpha _2$, then every $\alpha _2$-approx. min-cut 3-respects a spanning tree in $\mathcal {T}$,
- If $\alpha = \alpha _3$, then every $\alpha _3$-approx. min-cut 2-respects a spanning tree in $\mathcal {T}$.

The proof of Theorem 10 is a straightforward adaptation from Karger [11] (also see  [12]).

Since we can change the task of enumerating all $\alpha$-approximate cuts to that of enumerating over all 2, 3 or 4-respecting cuts of a collection of spanning trees $\mathcal {T}$, we need to show how to enumerate such respecting cuts efficiently. First, we compute the collection of spanning trees $\mathcal {T}$ using the algorithm in Theorem 10 with parameter $\alpha$. For each tree *T*:

- If $\alpha = \alpha _1$, then we enumerate all possible combinations of $(e_1,e_2,e_3,e_4)$ tree edges where $e_2,e_3,e_4$ could possibly be empty. The number of combinations is $O(n^4)$.
- If $\alpha = \alpha _2$, then we enumerate all possible combinations of $(e_1,e_2,e_3)$ tree edges where $e_2,e_3$ could possibly be empty. The number of combinations is $O(n^3)$.
- If $\alpha = \alpha _3$, then we enumerate all possible combinations of $(e_1,e_2)$ tree edges where $e_2$ could possibly be empty. The number of combinations is $O(n^2)$.

Let *U* be a set of edges drawn from tree *T* in the enumeration process, and *C* the corresponding cut induced by *U* in *G*. We denote *U* as $[[C]]_T$ to represent the encoding of *C* via the tree *T*. By Theorem 10, every $\alpha$-approximate min-cut *r*-respects some tree in the tree packing $\mathcal {T}$ for some $r \le 4$ with high probability. Then, every $\alpha$-approximate min-cut is listed in the enumeration with high probability.

However, during the enumeration, we need to compute the value $w(C\setminus F)=w(C) - w(F)$ where *F* is the set of *b* heaviest edges in *C*. Naively, this takes *O*(*m*) time per iteration. To speed up this computation, we preprocess each tree in the tree packing so that for any cut $[[C]]_T$ represented by the tree, we can compute $w(C \setminus F)$ in $\tilde{O}(b)$ time. This data structure is formalized in the following:

##### Lemma 5

(Cut Oracle) There is a data structure $\mathcal {O}$ that supports the following operations.

- $\mathcal {O}.\textsc {Construct}(G = (V,E,w),T,\gamma )$ where *G* is a weighted graph, *T* is a spanning tree of *G*, and $\gamma > 0$ is an integer: Initialize and preprocess the data structure in $\tilde{O}(\gamma ^2\cdot m)$ time.
- $\mathcal {O}.\textsc {Weight}([[C]]_T)$ where $|[[C]]_T| \le \gamma$: Return *w*(*C*) in $\tilde{O}(\gamma ^2)$ time.
- $\mathcal {O}.\textsc {kFreeWeight}([[C]]_T,k)$: Return $w(C \setminus F)$ where $F \subseteq C$ is the set of *k* heaviest edges in *C* in $\tilde{O}(\gamma ^2k)$ time.
- $\mathcal {O}.\textsc {Cut}([[C]]_T)$: returns the cut *C* in *O*(*m*) time.

We prove Lemma 5 in Section 4.2.3. This fact extends from known techniques, e.g., see [12]. In [12], they proved the case where $\gamma \le 2$, and we extend it to work for all $\gamma$.

#### The implementation

We are now ready to describe the fast implementation. Let us now look more closely at the fast implementation of Algorithm 2:

In Step 1 of Algorithm 2, the value ${\textsf{OPT}}^N$ can be $(1+\epsilon )$-approximated using the algorithm by [13] in $\tilde{O}(\frac{1}{\epsilon } \cdot m)$ time.

It remains to describe the fast implementation of Steps 2 and 3 of Algorithm 2. Recall that at this step, we are given $\tilde{w}$, a weight function on the input graph $G = (V,E)$. This differs from the input weight function *w*. We are also given $\alpha \in \{\alpha _1,\alpha _2,\alpha _3\}$ and $\varepsilon < 1/100$ as parameters.

1. Call the algorithm in Theorem 10 using parameter $\alpha$ and weight function $\tilde{w}$ to compute the collection of spanning trees $\mathcal {T}$ in $\tilde{O}(m)$ time.
2. For each tree $T \in \mathcal {T}$,
  - Construct the cut oracle (Lemma 5) $\mathcal {O}_T$ given the tree *T* in $\tilde{O}(m)$ time.
  - For each $[[C]]_T$ in the enumeration (see the section 4.2.1 above), call $\mathcal {O}_{T}.\textsc {kFreeWeight}([[C]]_T,b)$ to obtain $w(C\setminus F)$ where $F \subset C$ is the *b* heaviest edges in *C*. If the value is the smallest so far, update the solution to $[[C]]_T$.
3. Let $[[C']]_{T'}$ be a solution with the smallest value of $\mathcal {O}_{T'}.\textsc {kFreeWeight}([[C']]_{T'},b)$ among all trees in $\mathcal {T}$. We then extract $(C',F')$ as follows. First, compute $C' = \mathcal {O}_{T'}.\textsc {Cut}([[C']]_{T'})$ and $F'$, which is the *b* heaviest edges in $C'$.

***Analysis.***

The correctness follows immediately from Theorem 10 that every $\alpha$-approximate cut can be enumerated via tree packing and the correctness of the data structures in Lemma 5. We now analyze the running time. We run the algorithm by [13] to obtain an estimate $\tau$ in $\tilde{O}(\frac{1}{\varepsilon } \cdot m)$ time. Tree packing and data structure preprocessing can be done in $\tilde{O}(m)$ time. The number of iterations in the enumeration is $O(n^4), O(n^3), O(n^2)$ for $\alpha = \alpha _1, \alpha _2, \alpha _3$, respectively. The evaluation of the value $\tilde{w}(C \setminus F)$ from the implicit representation of the cut $[[C]]_T$ can be done in $\tilde{O}(b)$ time. In total, it takes $\tilde{O}(n^4b), \tilde{O}(n^3b), \tilde{O}(n^2b)$ time for $\alpha = \alpha _1, \alpha _2, \alpha _3$, respectively.

#### Proof of Lemma 5

This section is devoted to proving Lemma 5, which is a straightforward extension of the construction in [12] using the Euler tour tree and range tree data structures. To implement the operation $\mathcal {O}.\textsc {kFreeWeight}$, we use priority queues on top of the data structures provided by [12] to extract the top *b* edges efficiently.

We start by giving some preliminaries and definitions, and we then prove in Lemma 6 our extension to the data structure in [12] by first giving an overview of their construction and then explaining its generalization. At the end of the section, we explain how to use Lemma 6 to prove Lemma 5 by using an additional max-heap data structure.

***Notation.***

For convenience, when we say a cut we mean a set of edges $E(A,V\setminus A)$ for some vertex set *A* such that $\emptyset \ne A \subsetneq V$. For any graph *G*, we denote *V*(*G*) and *E*(*G*) as the set of vertices and edges in *G*, respectively. Let $G=(V,E)$ be a graph and let *T* be a spanning tree of *G*.

Recall Definition 4 for *r*-respecting cuts.

##### Definition 4

*(r*-*respecting cut)* Let $G=(V,E)$ be a graph and let *T* be a spanning tree of *G*. We say that a $(k-)$cut *C* in *G* *r*-respects *T*, if $|C\cap E(T)| = r$.

##### Definition 5

Given a spanning tree *T* with a fixed an root vertex $s \in V(T)$, the vertex set corresponding to the cut induced by a set of tree edges $F \subseteq E(T)$ is $S := \{v \in V(T) :|E(P_{s,v}) \cap F| \text { is odd} \}$, where $P_{s,v}$ is the unique path from *s* to *v* in *T*. We say that the cut $\delta _G(S)$ is *induced* by the set of *r* tree edges.

**Fig. 1:**
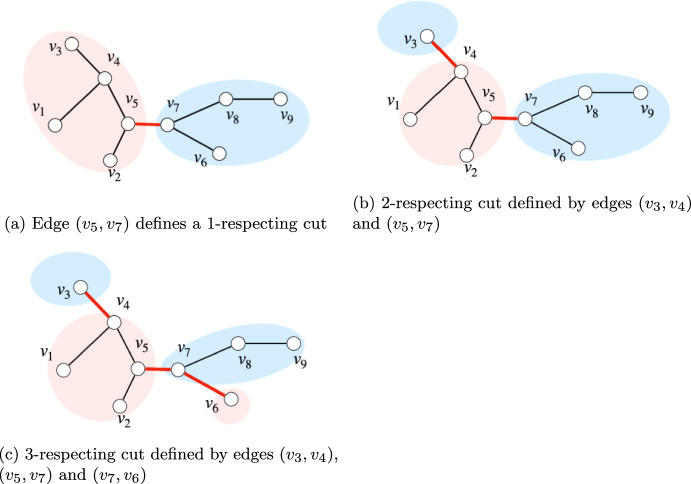
A spanning tree *T* and examples of 1, 2 and 3-respecting cuts defined by the removal of 1, 2 and 3 edges of the tree

See Fig.1 for examples of cuts induced by the removal of 1, 2 and 3 edges of a spanning tree.

***Tree packing and cut oracle.***

Let *T* be a spanning tree. If *C* is a cut that *t*-respects the tree *T* for some $t \le \gamma$, we denote $[[C]]_T := E(T) \cap C$, a set of tree edges in *T* that induce the cut *C*. We extend the canonical cuts data structure presented in [12] to support up to $\gamma$-respecting cuts.

##### Lemma 6

There is a data structure $\mathcal {D}$ that takes as inputs a weighted graph $G = (V,E,w)$, a spanning tree *T* of *G*, and supports the following operations.

- $\mathcal {D}.\textsc {Preprocess}(G,T)$: preprocess the inputs and return $\mathcal {C}$ a family of edge sets $D \subseteq E$ called *canonical cuts* such that $\sum _{D \in \mathcal {C}} |D| = \tilde{O}(m)$. This operation takes $\tilde{O}(m)$ time.
- $\mathcal {D}.\textsc {Decompose}([[C]]_T)$ where we denote $|[[C]]_T| = r$: Return $D_1, \ldots , D_\ell \in \mathcal {C}$ such that $C = \bigsqcup _{i \le \ell } D_i$ and $\ell \le \tilde{O}(r^2)$ where $\bigsqcup$ denotes the disjoint union operation. This operation takes $\tilde{O}(r^2)$ time.

The operation $\mathcal {D}.\textsc {Decompose}$ is ready to use after the operation $\mathcal {D}.\textsc {Preprocessing}$ is called.

##### Proof

The construction of this data structure is as follows:

***Euler tours.***

Let *T* be a rooted spanning tree of *G* on $V(G)=n$ vertices. We begin by computing an Eulerian Tour of *T* starting from the root *s*. We delete from the Eulerian tour all occurrences of a node *v* that are not the first or the last occurrences in the tour, and we then replace the first occurrence of node *v* by the labelled node $v^-$ and its last occurrence by the labelled node $v^+$. We denote the obtained sequence of labelled nodes by $\mathcal {S}$.

***Range Trees.***

We build a range tree $\mathcal {R}$ from $\mathcal {S}$ as follows. Place the labeled vertices of $\mathcal {S}$ as leaves in a tree and build a natural balanced tree out of these leaves by creating internal nodes denoted by the set $\mathcal {I}$. Each such node $a \in \mathcal {I}$ induces a unique interval in sequence $\mathcal {S}$ defined by the sequence of leaves in the sub-tree rooted at node *a* in $\mathcal {R}$.

***Canonical Intervals.***

We refer to an interval in $\mathcal {S}$ as *canonical*, whenever it corresponds to a sequence induced by the sub-tree rooted at a node in $\mathcal {R}$. Note that there exist *O*(*n*) many such canonical intervals and we denote the set of all of them by $D_I$.

##### Claim 1

Take any interval *I* in $\mathcal {S}$ (not necessarily canonical), then *I* can be decomposed into the sequence of at most $O(\log n)$ canonical intervals from $D_I$.

##### Proof

Let $I = (w_1, ... , w_f)$ be an interval where the $w_i's$ are labelled nodes in $\mathcal {S}$. Take the path $p_1$ in $\mathcal {R}$ from $w_1$ to the root. Assume that $w_1$ is on the left sub-tree (equivalently, right sub-tree) of the root. Take as canonical intervals all the sub-trees of all right (left) children of nodes in $p_1$ which are not themselves in $p_1$. We do the same for $w_f$ and take the disjoint union of the intervals found. Note that this ensures that the canonical intervals chosen are disjoint and at the same time that all vertices $w_i \in \mathcal {S}$ are contained in a canonical interval. Since $p_1$ contains $O(\log n)$ nodes in $\mathcal {R}$, this gives a decomposition into at most $O(\log n)$ many canonical intervals. $\square$

Note further, that one can similarly decompose into $O(\log n)$ canonical intervals the union and the complement of the union of any constant number of intervals in $\mathcal {S}$.

***Canonical cuts.***

Let $I_1, I_2$ be two canonical intervals, then we denote by $\mathcal {C}$ the set of all edges (*u*, *v*) in *E*(*G*) such that the labeled nodes $u^-$ and $v^-$ appear one in $I_1$ and the other in $I_2$. We call the set $\mathcal {C}$ a *canonical cut* induced by $I_1$ and $I_2$. Note that each labelled endpoint of an edge can be contained in at most $O(\log n)$ canonical intervals (since the height of $\mathcal {R}$ is $O(\log n)$) and hence each edge can be contained in $O(\log ^2 n)$ canonical cuts.

We are now ready to show how to compute the operations $\mathcal {D}.\textsc {Preprocess}(G,T)$ and $\mathcal {D}.\textsc {Decompose}([[C]]_T)$.

For the operation $\mathcal {D}.\textsc {Preprocess}(G,T)$, note that one can calculate the set of canonical cuts in $\tilde{O}(m)$ time as follows: First, calculate all the canonical intervals of *T* by computing an Eulerian tour of *T*, constructing its corresponding range tree $\mathcal {R}$ of size *O*(*n*) in $\tilde{O}(m)$ time and by looking at the subtree of each node in $\mathcal {R}$. Second, for every such pair of canonical intervals, we can compute its set of induced non-empty canonical cuts in $O(m \log ^2 n)$ time as follows: note that as we argued before each edge $e \in E(G)$ appears in at most $O(\log ^2 n)$ canonical cuts, so there are at most $O(m \log ^2 n)$ nonempty canonical cuts so we can construct them by adding each edge *e* to the $O(\log ^2 n)$ canonical cuts which contain it. This also shows that $\sum _{D \in \mathcal {C}} |D| = \tilde{O}(m)$. Note that our data structure stores both the set of canonical intervals and the set of canonical cuts, each identified by the pair of canonical intervals that induce it.

In [12], it is shown that all 1-or-2 respecting cuts of *T* can be decomposed into the disjoint union of $O({\log ^2 n})$ canonical cuts. For our purposes, we generalize the argument to show that every *r*-respecting cut can be decomposed into the disjoint union of $O(r^2 \log ^2n)$ canonical cuts.

Let *s* be a sequence of elements $w_1, ... , w_f$ and let us define the square brackets notation as follows:

- $[w_i,w_j]$ with $i\le j$ denotes the interval starting at and including element $w_i$ and ending at and including element $w_j$.
- $]w_i,w_j[$ denotes the interval starting at the element appearing immediately after and not including $w_i$ and ending at the element appearing immediately before and not including element $w_j$.
- $[w_i,w_j[$ denotes the interval starting at and including element $w_i$ and ending at the element appearing immediately before and not including element $w_j$.
- $]w_i,w_j]$ denotes the interval starting at the element appearing immediately after and not including $w_i$ and ending at and including element $w_j$.

**Fig. 2:**
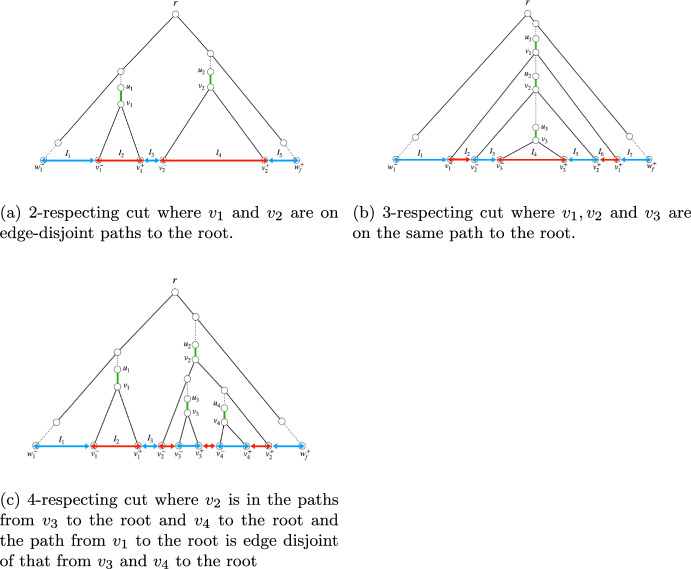
Examples for 2, 3 and 4-respecting cuts and their induced intervals. These intervals partition the leaf nodes into two sets, **red** and **blue**. An arrow head lays above a node when such node is included in the interval

***Decomposing*** *r*-***respecting cuts.***

Let $\mathcal {C}$ be a *r*-respecting cut of *T* that is induced by the removal of the edge set $F \subseteq E(T)$ where $|F| = r$. In the sequence $\mathcal {S}$, the removal of a tree edge $(u,v) \in E(T)$ defines a contiguous interval defined by the sub-tree rooted at $v \in T$, which is equivalent to the interval $I_v = [v^-, ..., v^+]$ in $\mathcal {S}$. Observe that the set of all intervals $\{I_v :v \in V(T)\}$ forms a laminar family. Let $\mathcal {I}_F = \{I_v :(u,v) \in F\}$ be the set of intervals defined by the edge removal *F*. Recall by Definition 5 that the vertex set corresponding to the cut induced by *F* is $S := \{v \in V(T) :|E(P_{s,v}) \cap F| \text { is odd} \}$, the set of vertices *v* whose unique path from the root *s* to *v* contains an odd number of edges in *F*. As a result, a vertex *v* belongs to the set *S* if and only if $v^-$ and $v^+$ are contained in an odd number of intervals in $\mathcal {I}_F$ (see Fig.2 for an illustration). Therefore, we can subdivide the entire sequence $\mathcal {S}$ into $O(|\mathcal {I}_F|) = O(r)$ subintervals *X* such that every interval is contiguous and every node in each interval is contained by the same number of intervals in $\mathcal {I}_F$. Among these *O*(*r*) subintervals *X*, we split them $X = Y \cup Z$ as either odd or even, depending on the parity of the intervals in $\mathcal {I}_F$.

For each interval $y\in Y$, we decompose each interval *y* into a set of $O(\log n)$ canonical intervals by Claim 1. Let $Y'$ be the union of all canonical intervals obtained from all $y \in Y$. We define $Z'$ similarly. This means we can compute the set of edges of *r*-respecting cut $\mathcal {C}$ by computing all the canonical cuts between all pairs of canonical intervals between $Y'$ and $Z'$. The number of canonical cuts for the *r*-respecting cut $\mathcal {C}$ is at most

$$|Y'|\cdot |Z'| \le O(\log ^2n) \cdot |Y|\cdot |Z| \le O(\log ^2n) \cdot O(\max _{1\le a < r} a\cdot (r-a)) = O(r^2\log ^2n).$$

The first inequality holds since each interval can be represented by $O(\log n)$ canonical intervals. The second inequality follows because *Y* and *Z* are disjoint and $Y \cup Z = X$. $\square$

Let us now show how to use Lemma 6 to construct the cut oracle data structure of Lemma 5.

##### Proof of Lemma 5

We use the data structure from lemma 6 and additionally store the weights of edges in each canonical cut so that retrieving the *b* edges of maximal weight in each of them can be done efficiently.

Let $\mathcal{C}\mathcal{C}=(I_1, I_2)$ be the set of edges in a canonical cut induced by the pair of canonical intervals $I_1$ and $I_2$. For each such canonical cut, we will create a max-heap $\mathcal {H}_{(I_1, I_2)}$ with keys equal to the weights of the edges in $\mathcal{C}\mathcal{C}$, that is, we construct $\mathcal {H}_{(I_1,I_2)}$ by inserting *w*(*e*) into the heap every time a new edge *e* is added into the canonical cut *CC*. Notice that the construction of such max-heap can be implemented in linear time.

Also note that we can easily compute the total weight of each canonical cut while preprocessing each canonical cut and we can store its value together with its corresponding canonical cut. Then, given a cut $[[C]]_T$, we can compute $\mathcal {O}.\textsc {Weight}([[C]]_T)$ by computing the sum of the weights of all canonical cuts in which cut $[[C]]_T$ decomposes by the function $\mathcal {D}.\textsc {Decompose}([[C]]_T)$ of lemma 6.

For $\mathcal {O}.\textsc {kFreeWeight}([[C]]_T,k)$, we do as follows: for each of the canonical cuts that $[[C]]_T$ decomposes into, we look at its max-heap and query its maximum (in *O*(1) time). We then select the maximum value from all the $\max$ queries to each canonical cut’s max-heap and pop that value from the max-heap which contained it and store it in a variable *k*-sum. This ensures that the maximum weight in cut $[[C]]_T$ is removed. This process takes $\tilde{O}(r^2)$ time. We can iterate this process by popping the current maximum element among all the max-heaps and adding it to the current value of the variable *k*-sum. Note that after *k* iterations, *k*-sum contains the sum of the weights of the *k* heaviest edges in cut $[[C]]_T$, so that $\mathcal {O}.\textsc {kFreeWeight}([[C]]_T,k)$ is computed by $\mathcal {O}.\textsc {Weight}([[C]]_T) - (k$-sum). After *k*-sum is computed, we can re-insert back the elements which we popped out during the $\max$ queries (note that there will be *k* re-insertions). Since we repeat at most *k* iterations, the running time is $\tilde{O}(kr^2)$.

Finally, $\mathcal {O}.\textsc {Cut}([[C]]_T)$ returns the cut *C* by finding the connected components in *T* after the removal of the *r* edges in *C*, respecting it. This can be done via DFS traversal in *O*(*m*) time. $\square$

## Footnotes

1 $\tilde{O}(\cdot )$ hides poly-logarithmic factors.

2 we will discuss how to compute $w^{(1)}$ efficiently later.

3 Recall that we assume that there is no cut *C* with $w(C) \le b$. Therefore, all of the $\lambda _i$s have value at least 1.

4 We note that in all the proofs in Section 2, the fact that the cut involved separates the graph into 2 or *more* components does not matter.
